# Excision Repair Cross-Complementation Group 1 Enzyme as a Molecular Determinant of Responsiveness to Platinum-Based Chemotherapy for non Small-Cell Lung Cancer

**DOI:** 10.4137/bmi.s485

**Published:** 2008-04-17

**Authors:** Giannis Mountzios, Meletios-Athanasios Dimopoulos, Christos Papadimitriou

**Affiliations:** Medical Oncology Unit, Department of Clinical Therapeutics, University Hospital Alexandra, Athens, Greece

**Keywords:** non small-cell lung cancer, ERCC1, predictive, biomarkers, platinum-based chemotherapy

## Abstract

Although platinum-based chemotherapy remains the “standard” in advanced non small-cell lung cancer, not all patients derive clinical benefit from such a treatment. Hence, the development of predictive biomarkers able to identify lung cancer patients who are most likely to benefit from cisplatin-based chemotherapy has become a scientific priority. Among the molecular pathways involved in DNA damage control after chemotherapy, the nucleotide excision repair (NER) is a critical process for the repair of DNA damage caused by cisplatin-induced DNA adducts. Many reports have explored the role of the excision repair cross-complementation group 1 enzyme (ERCC1) expression in the repair mechanism of cisplatin-induced DNA adducts in cancer cells.

Using immunohistochemistry in resected tumors from patients included in the International Adjuvant Lung Cancer Trial, the study of important biomarkers showed that high ERCC1 protein expression was associated with improved survival in chemo-naïve patients. On the contrary, the benefit of adjuvant cisplatin-based chemotherapy was more profound in patients with low ERCC1 expression. In a prospective cohort studying mRNA expression in tumor biopsies from patients receiving customized therapy with cisplatin and gemcitabine depending on the molecular profile of the tumour, results showed that patients with low ERCC1 mRNA expression had a longer median survival compared to those with high expression. These data suggest the potent use of ERCC1 as a molecular predictor of clinical resistance to platinum-based chemotherapy in the adjuvant setting of NSCLC. Nevertheless, optimization of methodology, including standardization of technical procedures, as well as validation of ERCC1 protein expression in large prospective cohorts, seem necessary before any routine immunohistochemical validation of ERCC1 can be implemented in daily practice.

## Introduction

Accounting for approximately 1,000,000 deaths every year and characterized by an increasing annual incidence of 0.5%, lung cancer remains the leading cause of cancer-related death worldwide [Spiro and Porter, 2002]. Response rates to chemotherapy for inoperable NSCLC vary from 30 to 60% for platinum combined with either gemcitabine or vinorelbine or a taxane. Although platinum-based chemotherapy regimens have demonstrated clinical activity against NSCLC, advanced disease remains fatal with short survival outcomes (approximately 5% at 5 years) [Dunant et al. 2005]. As a consequence, one of the most important challenges currently in medical oncology is to identify an optimal treatment strategy using individualized approaches. In the future, these will certainly be based on a precise knowledge of the tumor’s molecular signature [Blackhall and Thatcher, 2004].

Pre-clinical studies have already produced large amounts of information able to elucidate the biological mechanisms of cancer cell response to the stress of chemotherapy. From *in vitro* studies using lung cancer cell lines, more than 100 candidate genes have been identified as potent biomarkers of chemosensitivity [Sekine et al. 2006]. Those are genes implicated in physiological processes such as drug transport, drug metabolism, DNA repair, cell cycle regulation, and apoptotic or anti-apoptotic pathways. Among them, mechanisms of DNA repair play a crucial role in the preservation of genome integrity. They also allow to circumvent the damages induced by a large number of chemotherapeutic agents and thus contribute to clinical resistance to anticancer drugs. However, only few of the biomarkers related to DNA damage repair process are of clinical interest in lung cancer. Currently, the most extensively studied markers are ribonucleotide reductase M1 subunit (RRM1), which is implicated in the metabolism of nucleotides for DNA synthesis, p53 (implicated in the apoptotic response), and the excision repair cross-complementation group 1 (ERCC1), implicated in DNA repair. In the current review we describe the origin of the main DNA lesions, we refer to mechanisms of DNA repair and we focus on the role of ERCC1 as a molecular determinant of resistance to cisplatinum-based chemotherapy in NSCLC.

## DNA Lesions and Mechanisms of DNA Repair

**Figure 1:**
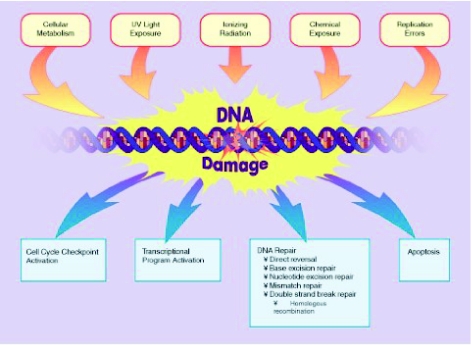
The main endogenous and exogenous mechanisms that can induce DNA damage (upper line), along with the main cellular mechanisms of DNA damage repair (lower line) Reproduced with permission.

DNA lesions are generally classified into three categories (Fig. 1): (1) Breaks of endogenous origin that include products of the cellular metabolism leading often to the spontaneous rupture of the glycosidic bond between the nucleosidic base and the deoxyribose, resulting in a simple break of DNA sequence which may, in rare cases, provoke irreversible damage and consequent cellular death [Friedberg, 2003]. A critical mechanism of endogenous DNA damage is the formation of free radicals produced in the mitochondrial respiration chain. Such molecules can directly react with the DNA molecule and form more than 100 different oxidative lesions [Beckman and Ames, 1997]. (2) Breaks of exogenous origin related to several environmental factors including the ultraviolet component of sunlight, which can induce the formation of dimers (DNA adducts). Some genotoxic substances contained in cigarette smoke (benzopyrenes) or in industrial waste are equally capable of provoking the formation of DNA adducts [Scharer, 2003]. (3) Finally, some anti-neoplastic drugs can generate DNA breaks depending on the mechanism of the action and the pharmacological properties of the molecule. These agents include the nitrosureas (carmustine, lomustine, fotemustine, streptozokine), the tetrazines (temozolamide, dacarbazine), the aziridines (thiotepa, mitomycin C), the bischloroethylamines (melphalan, chlorambucil) and notably the platinum combounds (cisplatin, carboplatin, oxaliplatin). The most frequent type of DNA damage inflicted by these agents is the formation of O^6^-methylguanine which can generate an irreversible error in DNA replication process, inevitably leading in cellular death [Bignami et al. 2000]. Finally, stabilisation of DNA topo-isomerases I and II by their corresponding inhibitors (camptothecins and epipodophyllotoxins) can result in simple or double chain break of the DNA molecule, inducing irreversible DNA damage [Wang, 1996].

In recent years, studies on mechanisms of chemotherapy resistance have focused on the identification of molecular markers involved in critical pathways through which the anti-neoplastic action of the drug is exerted. In the case of platinum compounds, the establishment of DNA lesions by covalent binding of platinum to the DNA molecule (commonly mentioned as DNA “adducts”) constitutes cisplatin’s main cytotoxic mechanism. Unfortunately, some cancer cells are able to circumvent drug action through increased DNA-repair capacity. ERCC1 enzyme is a rate-limiting DNA repair protein in the NER pathway that is specifically in charge of removing DNA platinum adducts [Lindahl and Wood, 1999; Mu et al. 1996; Sancar, 1994; Zamble et al. 1996].

**Figure 2:**
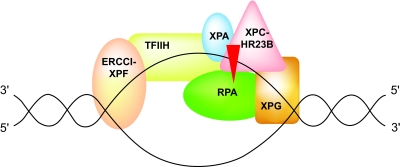
The main molecules that participate in the necleotide excision repair (NER) process. ERCC1: Excision repair cross-complementation group 1, XPA: *Xeroderma pigmentosum A* DNA helicase, XPF and XPG: *Xeroderma pigmentosum* F and G DNA endonucleases. TFIIH, RPA and XPC-HR23B: Molecular complexes for the recognition of the lesion and the incision of the damaged nucleotide. Reproduced with permission.

The NER pathway contains several steps, including the recognition of the lesion, opening of the double DNA helix around the damage, excision of the DNA strand carrying the platinum adduct, and ultimately polymerization of a new DNA strand followed by its ligation [Mu et al. 1996]. The NER process comprises at least 16 different proteins, including the XP proteins (groups A to G), the deficiency of which provokes a rare syndrome called *xeroderma pigmentosum (XP)*, characterized by hypersensitivity to ultraviolet radiation and a predesposition to skin cancer [Evans et al. 1997]. The NER pathway consists of two distinct molecular processes: The GG-NER (Global Genome *NER*), which repairs DNA lesions independently of their localisation in the genome, and the TC-NER (*Transcription-Coupled NER)* pathway, which is activated by lesions in DNA regions involved in transcription (transcriptomes). In both cases, the unfolding of the double DNA helix is assured by the helicases XPD (polarity 3′-5′) and XPB (polarity 5′-3′) [Scharer, 2003]. This process renders the lesion accessible to the endonuclease XPG, which, in conjunction with the helicase XPA, recognizes and verifies the presence of the DNA lesion and subsequently performs the incision of the damaged nucleotide in the 3′ edge of the lesion (Fig. 2). In a third critical step, the endonuclease XPF, in association with ERCC1, removes the damaged nucleotide from the 5′ edge of the damaged chain and deliberates a fragment of 24–32 bases [Fiedberg, 2001; Hoeijmakers, 2001]. Finally, a complex of DNA polymerases and ligases are recruited in order to perform the synthesis of missing complementary nucleotides and restore normal nucleotide sequence in the damaged chain [Fiedberg, 2001; Hoeijmakers, 2001].

### ERCC1 and DNA damage repair

In human cells, ERCC1 is a 15-kb gene located on the chromosome arm 19q and encodes for a nuclease of about 33 kDa. ERCC1 has been highly preserved during evolution and is constitutively expressed in all tissues at relatively high levels. The knock down of the ERCC1 gene in mice leads to an accelerated ageing phenotype with brain damage, liver failure and death shortly after weaning [Nunez et al. 2000]. Importantly, the ERCC1 protein is one of the rate-limiting enzymes in the NER complex [Volker et al. 2001], together with its obligate partner, the xeroderma pigmentosum group F (XPF) protein. A correlation between the expression of ERCC1 and resistance to platinum compounds has been consistently suggested in patients with advanced-stage gastric, ovarian, colorectal, esophageal and non small-cell lung cancer [Altaha et al. 2004; Dabholkar et al. 1992; Dabholkar et al. 1994; Langer et al. 2005; Joshi et al. 2005; Reed et al. 2000; Metzger et al. 1998; Lord et al. 2002; Rosell et al. 2002; Shirota et al. 2001; Warnecke-Eberz et al. 2004].

### ERCC1 as a risk stratifier in non small-cell lung cancer

A decade ago, a world-wide individual data-based meta-analysis [NSCLC collaborative group, 1995] suggested that cisplatin-based chemotherapy improved overall survival in advanced non small-cell lung cancer (NSCLC). Since then, several trials in the adjuvant setting have been conducted and recent results showed that platinum-based treatment improves survival by 4%–15%, depending on the TNM stage of the disease at diagnosis, among patients with surgically resected NSCLC [Scagliotti et al. 2003; Douillard et al. 2006; Winton et al. 2005; IALT collaborative group, 2004; Le Chevalier et al. 2005; Strauss et al. 2004]. Among these trials, the International Adjuvant Lung Cancer Trial (IALT) that included more than 1,800 patients demonstrated a 14%-decrease in individual risk of death and a 4.1% absolute benefit in 5-year overall survival for patients treated with adjuvant cisplatin-based chemotherapy [IALT collaborative group, 2004]. Although platinum-based chemotherapy presently remains the “standard” in advanced NSCLC, not all patients derive substantial clinical benefit from such a treatment. As a consequence, the IALT-bio study group prospectively collected tumor specimens from the IALT patients in an effort to identify molecular biomarkers that could determine responsiveness to platinum-based chemotherapy. In this recent work, findings concerning ERCC1 immunohistochemical expression on 761 tumors from the IALT study were reported [Olaussen et al. 2006]. ERCC1 immunohistochemical expression in the tumour samples was dichotomized to either “high” or “low”, using the median value of ERCC1 expression as the “cut off” value. Consistent with the above-mentioned studies in other solid tumours, high ERCC1 expression was correlated with better prognosis in patients that had not received chemotherapy. On the other hand, an inverse correlation was identified when patients were treated with cisplatin-based chemotherapy, meaning that the benefit from adjuvant chemotherapy was more profound in patients with low ERCC1 expression. It was concluded that NSCLC patients with completely resected ERCC1 negative tumors seem to derive a substantial benefit from adjuvant cisplatin-based chemotherapy compared to those with resected ERCC1 positive tumors.

The above-mentioned conclusion is consistent with recent findings from other studies: Zhou et al. [Zhou et al. 2004] demonstrated that an increased number of variant alleles in ERCC1 that render the molecule less efficient was associated with a decreased overall survival in patients with advanced NSCLC treated with platinum agents and that the number of these alleles could be predictive for overall survival. Isla et al. [Isla et al. 2004] studied a single nucleotide polymorphism in ERCC1 in peripheral blood lymphocytes from patients with advanced disease treated with cisplatin-based chemotherapy and concluded that patients with tumours bearing the polymorphism had a significantly better survival. Moreover, Rosell and colleagues performed different studies examining the role of both ERCC1 and RRM1 mRNA expression in paraffin-embedded pretreatment bronchial biopsies from patients with advanced NSCLC [Rosell et al. 2006; Rosell et al. 2003; Rosell et al. 2004]. Low ERCC1 mRNA levels were associated with better median survival in patients who received cisplatin-based chemotherapy. Bepler et al. [Bepler et al. 2006] recently reported an analysis of RRM1 and ERCC1 gene expression in relation with tumor response. Their results suggest that ERCC1 and RRM1 expression evaluated by real-time RT-PCR are predictors of tumor response in patients treated with the gemcitabine-platinum doublet regimen. In another retrospective analysis of 70 selected formalin-fixed, paraffin-embedded lung tumour biopsies, Ceppi et al. [Ceppi et al. 2006] studied patients treated either with the combination of cisplatin and gemcitabine, or with gemcitabine alone. They also reported that patients presenting low ERCC1 tumor expression had a longer median survival than those with high ERCC1 expression. Finally, in a recent retrospective study [Azuma K et al. 2007], immunohistochemical expression of ERCC1 was able to predict progression-free and overall survival in patients receiving platinum-based chemotherapy for recurrent tumours after curative resection. Similar conclusions have been recently reported for limited-stage small-cell lung cancer (SCLC) treated with platinum-based chemotherapy [Lee et al. 2007]. A summary of the clinical trials associating ERCC1 function with response to platinum-based chemotherapy in NSCLC patients is presented in Table 1.

### Clinical prospective trials on ERCC1

After the encouraging results of a prospective phase II study customizing chemotherapy according to ERCC1 gene expression [Simon G et al. 2007], Cobo et al. recently published the results from a prospective phase III randomized trial [Cobo et al. 2007], customizing cisplatin administration based on quantitative ERCC1 mRNA expression in tumours from patients with metastatic disease. The authors hypothesized that patients receiving therapy according to their baseline tumor ERCC1 mRNA levels would achieve higher response rates and more prolonged survival than patients in the control arm receiving non-customized therapy. Patients in the control arm received docetaxel plus cisplatin, while patients in the experimental arm received either the same combination, if their ERCC1 mRNA levels were low, or docetaxel plus gemcitabine, if their ERCC1 levels were high. Among the 444 patients enrolled in the study, 346 patients were evaluable for response. Objective response rates were 39.3% in the control arm and 50.7% in the experimental arm (p = 0,027). This study showed for the first time that assessment of ERCC1 mRNA expression in tumour tissue is feasible in the clinical setting in order to predict response to platinum-based chemotherapy (Table 1).

## Applicability of ERCC1 Measurements in Clinical Practice

Current data suggest that ERCC1 is a potentially useful marker for predicting clinical resistance to platinum compounds such as cisplatin, carboplatin and oxaliplatin. However, no study has yet provided robust evidence to consider ERCC1 as a marker for resistance to all types of chemotherapy. Before the potential of ERCC1 as a predictor of chemosensitivity can be fully evaluated, several technical and practical obstacles should be addressed.

First, the diagnosis is made from small tumor samples (biopsies) with limited quantity of bronchial tissue. Since ERCC1 immunostaining is characterized by heterogeneity [Olaussen et al. 2006], suboptimal sampling might mean that the biopsy is not necessarily representative of the whole tumour. Furthermore, tumour material could be obtained either from the primary or the metastatic sites. This may be significant since it has not been shown that ERCC1 expression is identical in the primary tumour and its metastatic locations.

Secondly, studies associating ERCC1 with resistance to platinum compounds have been so far conducted mainly by DNA or RNA molecular analysis, which is both technically demanding and expensive. The IALT-bio study had the advantage of using immunohistochemistry to assess ERCC1 expression, which is a feasible technique in everyday practice of a pathology laboratory. Should the technical aspects of ERCC1 immunohistochemistry become standardized and optimized, this methodology could rapidly be translated into a clinical reality after other confirmatory prospective studies. Currently, however, difficulties including the identification of an optimal “cut-off” value for ERCC1, the subjectivity of the pathological examination, particularly in prospective studies, and the heterogeneity of staining, that renders the technique of tissue micro-arrays rather inapplicable, limit the clinical usefulness of such procedures. Other difficulties, such as different genetic backgrounds in the population, or interaction with smoking habits, should be further discussed. In any case, meticulous methodological considerations and standardization are mandatory. It is necessary that future larger and prospective studies should be extensively discussed within multidisciplinary groups including highly skilled pathologists and bio-statisticians.

### Future perspectives and challenges

In the future, it seems probable that patients with ERCC1 positive tumours will be treated with non-platinum based regimens, whereas patients with ERCC1 negative tumours will be considered as optimal candidates for platinum-based therapy. In chemo-naïve patients, high ERCC1 expression is associated with a better prognosis since it provides the cell with a more competent DNA repair mechanism. Indeed, such a dual role of the ERCC1 protein is in agreement with recent proposals considering that DNA repair acts as a “double-edged sword” [Wei et al. 2000]. According to this theory, low DNA repair capacity allows for higher genomic instability within the cancer cell and consequently a faster acquisition of more aggressive malignant characteristics in chemo-naïve patients. On the other hand, at the same time it also predicts good response to DNA-targeting compounds because it deprives cancer cells from adequate DNA repair mechanisms. Globally, it also suggests the possibility that ERCC1 might have alternative functions in cancer cells. It is not yet known why ERCC1 is essential for life, whereas other limiting proteins in the NER repair pathway are not (such as XP patients carrying mutations in XPA, -B, -C, -D, -F, or G). There is clearly a need for a better understanding of the alternative functions of ERCC1, and further research in fundamental biology is highly awaited.

## Conclusion

Platinum-based chemotherapy improves survival of patients with non small-cell lung cancer, but not all patients derive therapeutic benefit from this treatment. Emerging evidence suggests the association of the molecular characteristics of the tumour with responsiveness to platinum-based chemotherapy. The DNA repair protein ERCC1, which is both a prognostic marker for survival and a predictor for response to platinum compounds, is currently an attractive molecular marker undergoing clinical testing in order to allow a more selective therapeutic strategy. Nevertheless, important issues regarding standardization and optimization of technical procedures for ERCC1 analysis in tumor samples remain to be resolved before this methodology can be implemented in daily clinical practice.

## Figures and Tables

**Table 1:** Main clinical trials associating ERCC1 function with response to platinum-based chemotherapy in NSCLC patients.

Author	N	Evaluation	Method of analysis	Conclusion
Olaussen et al. [29]	761	Retrospective	ERCC1 IHC expression	ERCC1 negative tumours derive benefit from cisplatin-based chemotherapy
Zhou et al. [50]	128	Retrospective	RT-PCR for ERCC1 polymorphisms (C8092A)	Increased number of variant ERCC1 alleles associated with decreased overall survival
Isla et al. [18]	62	Retrospective	RT-PCR for ERCC1 SNPs in mRNA from peripheral blood lymphocytes	Patients bearing the homozygous 118C allele have a significantly better survival
Rosell et al. [32–34]	100	Retrospective	RT-PCR for ERCC1 mRNA	Low ERCC1 mRNA levels associated with better median survival
Ceppi et al. [8]	70	Retrospective	RT-PCR for ERCC1 mRNA	Low ERCC1 mRNA levels associated with better median survival
Azuma et al. [2]	67	Retrospective	ERCC1 IHC expression	ERCC1 ICH expression is prognostic for survival after cisplatin-based chemotherapy
Bepler et al. [5]	54	Prospective phase II	RT-PCR for ERCC1 gene	ERCC1 expression predictive of tumor response
Simon G et al. [40]	60	Prospective phase II	RT-PCR for ERCC1 gene	ERCC1 expression predictive of tumor response
Cobo et al. [9]	444	Prospective phase III	RT-PCR for ERCC1 mRNA	ERCC1 mRNA levels can be used to customize therapy in the clinical setting
